# Bilateral Chronic Central Serous Chorioretinopathy Leads to Macular Atrophy: A Case Report

**DOI:** 10.7759/cureus.115772

**Published:** 2026-09-04

**Authors:** Othman Haddani, Noor R Jaber, Amine Razzak, Mohamed Bouazza, Mohamed Elbelhadji

**Affiliations:** 1 Ophthalmology, Mohammed VI University of Health Sciences (UM6SS), Casablanca, MAR; 2 Biology, King's InterHigh, London, GBR

**Keywords:** acetazolamide, cscr, macular atrophy, multimodal imaging, pachychoroid

## Abstract

We report a rare case of progressive bilateral atrophic maculopathy in the setting of a pachychoroid, secondary to chronic and complicated central serous chorioretinopathy (CSCR). A 58-year-old man reported progressive bilateral visual acuity loss and a central scotoma in the left eye, with a background of longstanding contralateral CSCR. Clinical examination and multimodal imaging (optical coherence tomography (OCT) and fluorescein angiography) confirmed subretinal fluid (SRF) in OD with atrophic maculopathy within the pachychoroid spectrum, without any indirect signs of active neovascularization. Treatment with oral acetazolamide and a topical anti-inflammatory agent was initiated, and the patient was closely monitored. During follow-up, visual acuity improved in the right eye, with a reduction in SRF, while these remained stable in the left eye. This case is notable for its asymmetric bilateral presentation, with unilateral atrophy and persistent contralateral subretinal serous detachment, a pattern rarely described in chronic CSCR. It also highlights that in cases where therapeutic options are limited, acetazolamide and anti-inflammatory drops can help reduce SRF and increase visual acuity. The development of new pseudodrusen and the absence of active exudation, despite advanced involvement, further underscore the atypical nature of this presentation.

## Introduction

Central serous chorioretinopathy (CSCR) is an ophthalmologic condition that affects the retina. This disease predominantly affects young and middle-aged adults and is characterized by accumulation of subretinal fluid (SRF) secondary to dysfunction of the retinal pigment epithelium (RPE) and the choroid [1]. There are three subtypes of CSCR: acute, recurrent, and chronic, depending on the duration of the SRF [2]. The chronic form is often defined by a duration of between three and six months of the SRF, even though this threshold is debated, and cases with persistent SRF are sometimes referred to as non-resolving CSCR [3]. Chronic CSCR can lead to visual loss, blurred vision, central scotomas, metamorphopsia, and micropsia [4]. Although CSCR was historically regarded as a primarily retinal disease, substantial evidence now supports a major contribution of choroidal and scleral abnormalities to its pathogenesis. In particular, choroidal vascular congestion, venous dilation, and increased vascular permeability have emerged as important features of the disease [5,6]. These clinical features fall within the pachychoroid spectrum of diseases, which are often characterized by increased choroidal thickness, dilated choroidal veins (pachyvessels), attenuation of the choriocapillaris, and retinal pigment epithelial changes, typically identified using enhanced depth imaging optical coherence tomography (OCT) and fundus autofluorescence [7]. This spectrum also includes more advanced manifestations, such as uncomplicated pachychoroid, pachychoroid neovasculopathy [8], pachychoroid aneurysmal type 1 neovascularization known as polypoidal choroidal vasculopathy (PCV), peripapillary pachychoroid syndrome [9], pachyvessels, pachydrusen, and focal choroidal excavation [10]. We present a case illustrating progressive bilateral atrophic maculopathy in a 58-year-old man on a background of chronic CSCR, with persistent unilateral serous detachment and contralateral macular atrophy. This case illustrates an atypical, advanced form confirmed by multimodal imaging and partially responsive to oral acetazolamide.

## Case presentation

A 58-year-old man with no history of diabetes, hypertension, or familial age-related macular degeneration (AMD) was admitted to the hospital on April 8, 2025, for progressive bilateral visual acuity loss, associated with a central scotoma in the left eye, presenting with a 10-year history of CSCR in the left eye. The patient has functional bowel disease and is on an anxiolytic medication, reflecting a chronic stress condition that may have influenced disease progression. Additionally, his ophthalmological examination revealed a visual acuity of 6/10 in the right eye (OD) and 2/10 in the left eye (OS); ocular tension was 16 mmHg in the right eye and 17 mmHg in the left eye (air tonometer). The anterior segment examination was unremarkable in both eyes. Fundus examination revealed papillary dysversion and an area of chorioretinal atrophy (CRA) involving the macula in both eyes. Fluorescein angiography performed on April 8, 2025, revealed early hyperfluorescence in the macular area corresponding to a window defect caused by RPE atrophy in both eyes, fading in the late phase, along with significant CRA (Figures 1-2).

**Figure 1:**
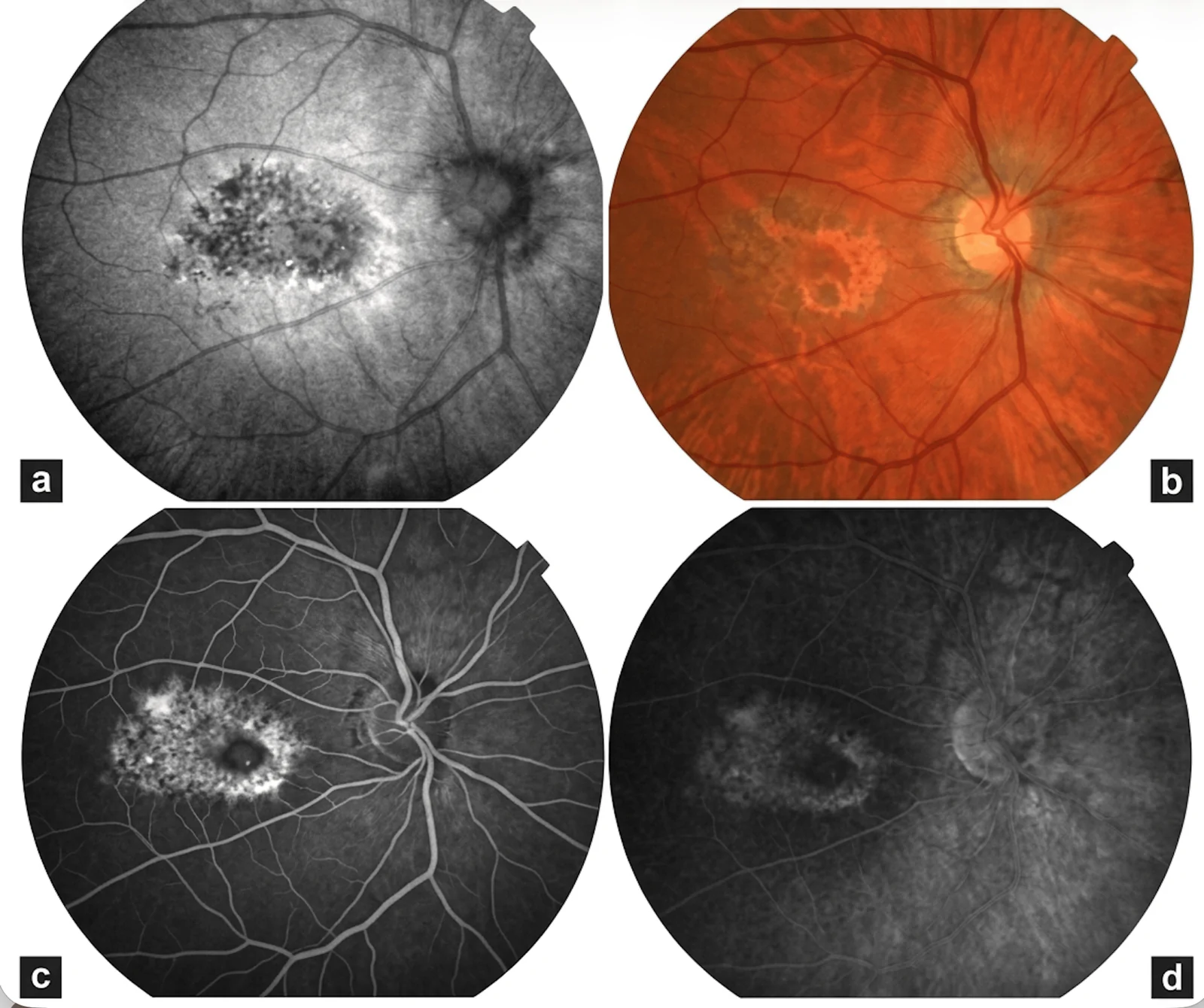
Fluorescein angiography of the right eye (OD) (a, b) Red-free and color fundus images showing papillary dysversion and significant chorioretinal atrophy (CRA). (c) Early phase: immediate central hyperfluorescence in the macular area. (d) Late phase: continued attenuation of the central hyperfluorescence signal, without diffusion or active leakage, consistent with a chronic atrophic form.

**Figure 2:**
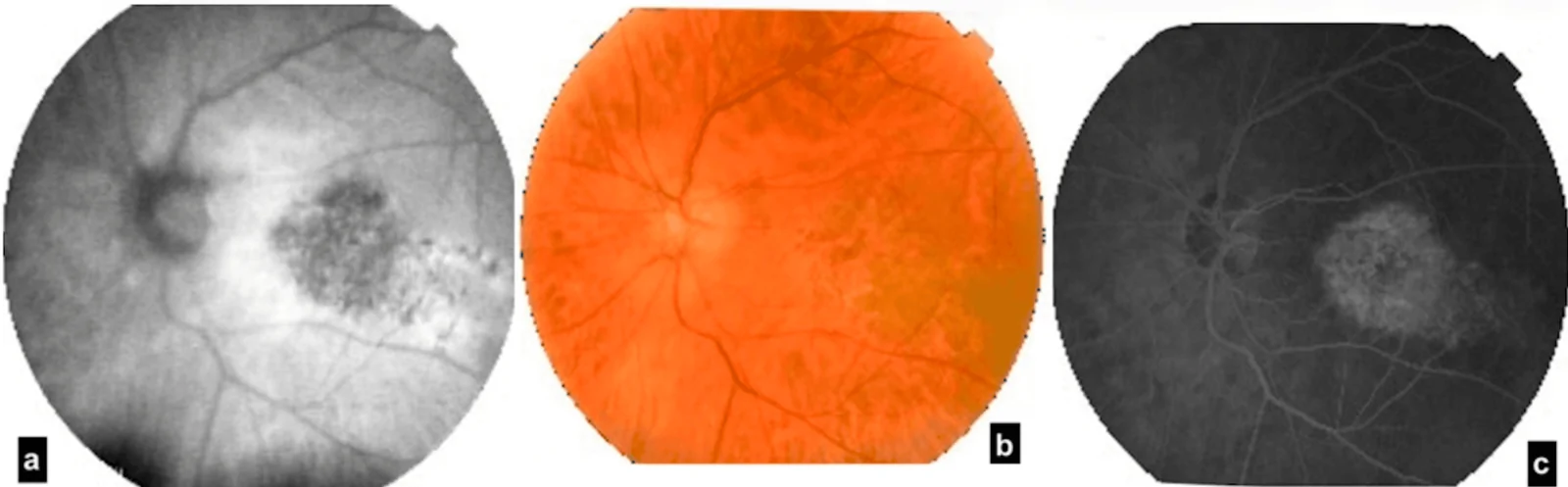
Fluorescein angiography of the left eye (OS) (a, b) Red-free and color images: significant central chorioretinal atrophy. (c) Early phase: immediate central hyperfluorescence in the macular region.

The OCT of the macula performed on April 10, 2025, demonstrated, in OD, an average central macular thickness of 273.1 µm. The macular atrophic changes were characterized by choroidal hypertransmission, RPE disruption, and overlying outer retinal degeneration, with active SRF of 159 µm; the underlying choroid appears thickened with prominent large-caliber choroidal vessels (Figure 3). In OS, the average central macular thickness was 247 µm; OCT demonstrated marked disruption and attenuation of the RPE, associated with focal loss of the ellipsoid zone and thinning of the outer retinal layers. Areas of increased choroidal signal transmission are visible beneath the altered RPE, consistent with RPE loss and outer retinal atrophy (Figure 3).

**Figure 3:**
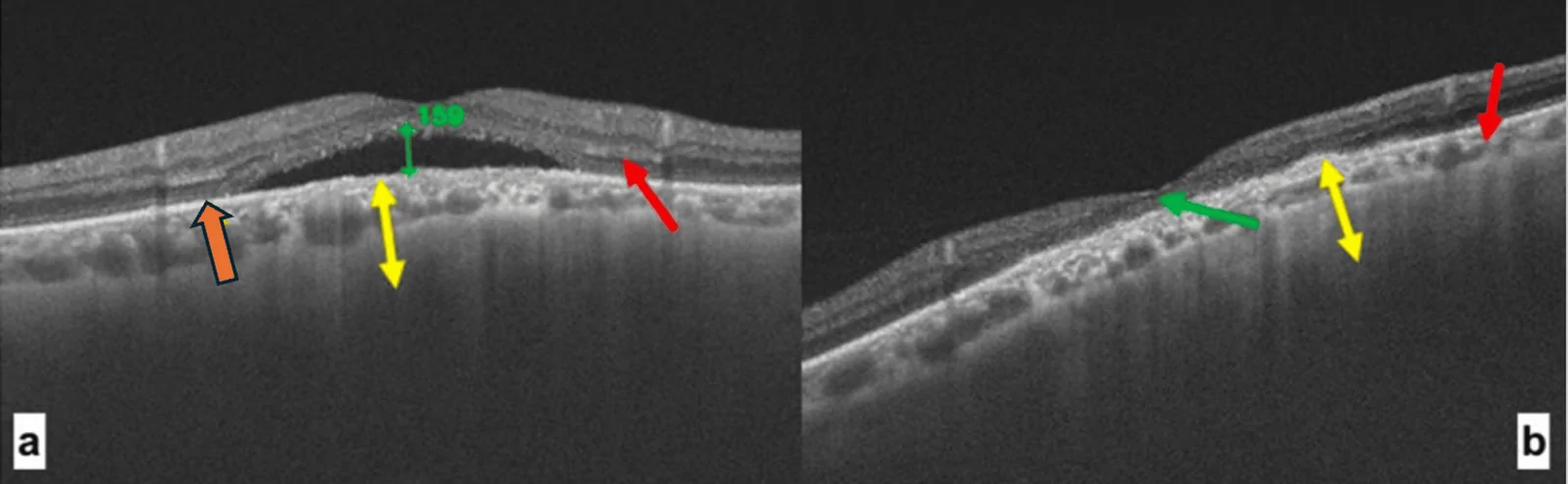
Macular OCT of the right eye (OD) and left eye (OS) ﻿﻿﻿﻿(a) OD: Macular SRF is present. The SRF height is measured at 159 μm, associated with choroidal thickening (yellow double-ended arrow), irregularities of the RPE (red arrow), and presence of drusen (orange arrow). (b) ﻿﻿﻿﻿OS: Macular atrophy (green arrow) with loss of the foveal pit, irregularity of RPE (red arrow), and thickening of the choroidal vessels (yellow double-ended arrow), without SRF. The vitreomacular interface is intact. OCT: optical coherence tomography; SRF: subretinal fluid; RPE: retinal pigment epithelium

The patient was treated with acetazolamide 250 mg tablets at a dose of half a tablet three times daily (125 mg every eight hours), corresponding to a total daily dose of 375 mg, for more than one month. Potassium chloride oral solution was added to the acetazolamide treatment to prevent hypokalemia. For local treatment, the patient was prescribed a topical nonsteroidal anti-inflammatory drug (NSAID) (indomethacin) at a dose of three eye drops per day for one month.

During follow-up, one month later, on May 11, 2025, there was an increase in visual acuity in the right eye to 7/10, and it was stable in the left eye. The macular OCT demonstrated a reduction to 240.5 µm in the average retinal thickness in OD, indicating partial SRF resorption, whereas OS remained stable at 242.7 µm, consistent with chronic atrophic changes. OCT showed a reduction in macular SRF, with persistent RPE irregularities and micro-remodeling, pseudo-reticular drusen, and an average choroidal thickness of 380 µm in OD and 245 µm in OS (stable compared with the initial OCT), with pachyvessels observed on OCT. The SRF decreased to 139 µm in OD (Figure 4). We kept the same treatment with acetazolamide for three months (same dose of 375 mg a day) and monitored the renal function during this time. The visual acuity was stable, and the OCT findings were the same as of May 11, 2025, with no significant changes.

**Figure 4:**
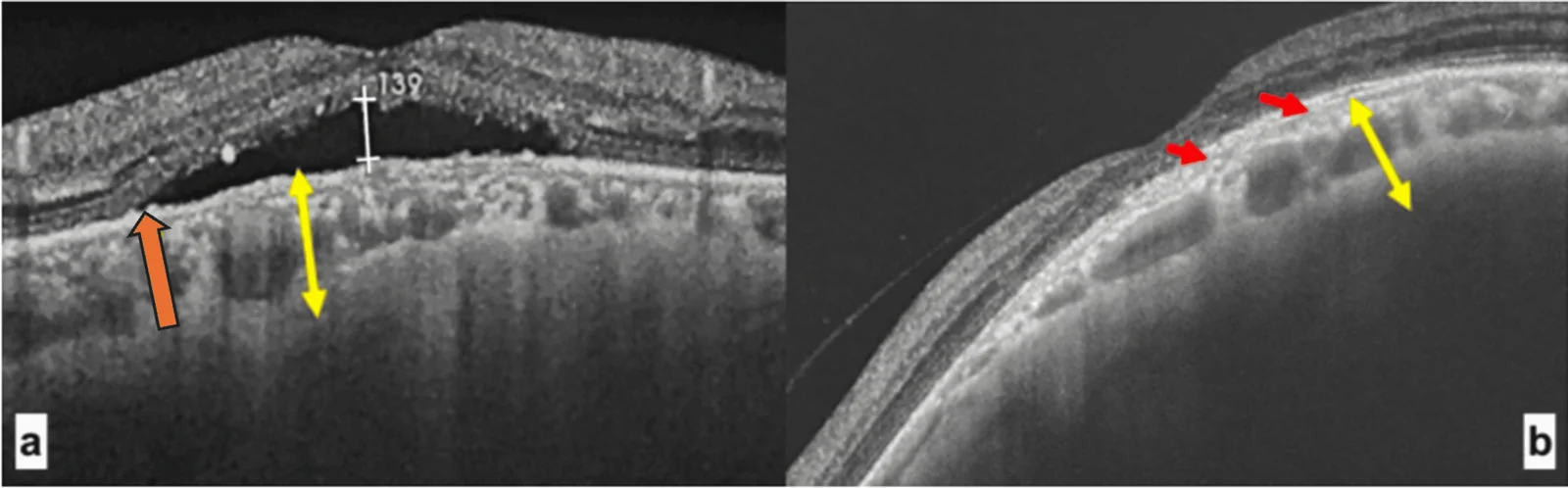
Evolution of macular OCT of the right eye (OD) and left eye (OS) one month later (a) ﻿﻿﻿﻿OD: partial regression of the SRF under treatment with oral acetazolamide, subretinal fluid reduced to 139 μm, appearance of reticular pseudo-drusen (orange arrow) and choroidal thickening (yellow double-ended arrow). (b) ﻿﻿﻿﻿OS: persistent atrophy, with increased RPE irregularities (red arrows) and stable choroidal vascular thickening (yellow double-ended arrow), with no SRF. There is no abnormality of the vitreomacular interface. SRF: subretinal fluid; RPE: retinal pigment epithelium

## Discussion

The incidence of CSCR is not well established and varies across studies. It has been reported to range from 9.9 per 100,000 men to 1.7 per 100,000 women [11]. The male-to-female ratio has been reported as approximately 2:1 by some authors and as high as 6:1 in other studies [12]. Chronic CSCR is often associated with progressive macular atrophy, particularly in severe or long-standing cases. Clinical studies have shown that diffuse atrophic changes of the RPE tend to increase over time, reflecting the progressive nature of the disease [13]. Within this framework, the pachychoroid concept helps explain the underlying structural alterations, including choroidal thickening, pachyvessels, and secondary RPE dysfunction. Enhanced-depth imaging optical coherence tomography (EDI-OCT) provides valuable characterization of choroidal alterations within the pachychoroid disease spectrum. Our patient exhibited progressive pachychoroid maculopathy, with persistent SRF in OD and severe atrophy in OS. OCT of OD revealed RPE irregularities with overlying reticular pseudodrusen, consistent with advanced pachychoroid-related changes. These findings are consistent with the expanded pachychoroid spectrum, which encompasses aneurysmal type 1 neovascularization, previously described as polypoidal choroidal vasculopathy (PCV) [14]. Importantly, multimodal imaging excluded active neovascularization in this case: no leakage or progressive hyperfluorescence on fluorescein angiography, consistent with a non-exudative form [15]. While acute CSCR often resolves spontaneously, with SRF clearance and relatively favorable visual outcomes, intervention may be warranted in cases of persistent or recurrent SRF, as well as in more complicated forms of CSCR, to reduce the risk of irreversible visual dysfunction [16]. Our case demonstrates a marked difference in the potential for reversibility between the two eyes. In the left eye, macular atrophy is already established and appears irreversible, whereas the right eye remains at a potentially salvageable stage, with retinal changes that may still be manageable. There are different treatment modalities in this pathology, such as oral medications, focal laser, photodynamic therapy (PDT), intravitreal injections, and surgery [4]. Focal laser photocoagulation targets and coagulates a localized leakage point responsible for SRF accumulation, as identified by fluorescein angiography. It is primarily considered a treatment option for acute or recurrent forms of CSCR. However, its efficacy in chronic CSCR is more limited, with less favorable and more variable outcomes [4]. PDT involves the intravenous administration of the photosensitizing agent verteporfin, which preferentially accumulates within the choroidal vasculature. A laser is then applied to the selected treatment area, activating the verteporfin molecules [4]. This photochemical reaction generates free radicals that induce a localized vascular response, including microthrombotic changes, inflammation, apoptosis, and occlusion of abnormal choroidal vessels. The resulting vascular remodeling is believed to decrease choroidal hyperpermeability and, consequently, reduce SRF accumulation [17]. Several studies have evaluated the efficacy of PDT in the management of CSCR. Among the most influential randomized controlled trials, the SPECTRA [18] and PLACE [19] studies provided important evidence supporting the use of PDT in patients with chronic CSCR. Acetazolamide is a carbonic anhydrase inhibitor. This class of medication has also been investigated as a potential treatment for chronic CSCR, based on its established effects on subretinal and intraretinal fluid in other RPE-related disorders [4]. Our patient was treated with oral acetazolamide to promote resorption of SRF, a common feature of chronic CSCR. Although evidence in chronic cases is limited, pilot studies and case reports in acute and recurrent CSCR suggest that acetazolamide can enhance RPE fluid transport and accelerate SRF resolution, providing a rationale for its use in persistent or long-standing fluid accumulation [20]. Given the patient’s persistent SRF and absence of active neovascularization, acetazolamide was selected to accelerate fluid resorption, as spontaneous resolution in chronic CSCR is often unpredictable. Additionally, potassium syrup was prescribed to mitigate potential hypokalemia associated with acetazolamide use. Topical nonsteroidal anti-inflammatory eye drops (Indomethacin) were also used to address local inflammation and may have contributed to improved SRF resorption, though their role remains adjunctive in chronic non-exudative CSCR. This combined approach led to partial regression of SRF in the right eye, supporting the therapeutic potential of these treatments in non-neovascular forms of CSCR. Moreover, these findings highlight the potential utility of acetazolamide in selected chronic cases despite limited large-scale evidence. Mineralocorticoid receptor antagonists, such as eplerenone and spironolactone, have been explored for chronic CSCR management. However, their efficacy remains unproven, and they are not approved for this indication. The VICI trial, a multicenter, randomized, double-blind, placebo-controlled study, found that eplerenone did not improve best-corrected visual acuity compared with placebo in patients with chronic CSCR after 12 months of treatment [21]. Given the unproven efficacy and lack of approval for mineralocorticoid receptor antagonists in CSCR, these agents were not included in the treatment regimen. Historically, these therapies have been investigated as alternatives to direct fluid resorption strategies, but evidence remains limited. Moreover, in our case, OCT measurements showed a reduction in average retinal thickness in the right eye from 273.1 µm to 240.5 µm. In contrast, the left eye remained stable at approximately 245 µm, consistent with chronic atrophy (Table 1).

**Table 1:** Summary table of the evolution of the case during follow-up SRF: subretinal fluid

Eye/visit	Visual acuity	SRF (µm)	Retinal thickness (µm)	Choroidal thickness (µm)
Right eye				
First visit	6/10	159	273.1	380
One-month follow-up	7/10	139	240.5	380
Left eye				
First visit	2/10	No fluid	247	245
One-month follow-up	2/10	No fluid	242.7	245

Differentiating CSCR-related atrophic changes from AMD is critical, particularly in older patients. Although the patient is in his late 50s, the absence of dense drusen, presence of pachyvessels, and localized choroidal thickening support a pachychoroid-driven pathology rather than AMD, informing prognosis and guiding management away from anti-vascular endothelial growth factor (VEGF) therapy. This case exemplifies a rare form of asymmetric bilateral involvement, where spontaneous or post-treatment regression of SRF remains unpredictable. While anti-VEGF therapy is indicated in neovascular forms, it is not justified here due to the absence of active neovascularization [22]. This underscores the importance of precise differential diagnosis using multimodal imaging to distinguish exudative from atrophic forms.

## Conclusions

This report describes a case of bilateral chronic asymmetric CRSC, with atrophic involvement in the left eye and progressive SRF accumulation in the right eye. Multimodal imaging was essential for accurate diagnosis and longitudinal monitoring. Chronic and advanced forms of CRSC may have limited therapeutic options compared with acute disease and require timely management to prevent progression toward macular atrophy, which may result in irreversible visual impairment. This therapeutic challenge is particularly relevant in countries where verteporfin is unavailable or difficult to access, while spironolactone has not demonstrated consistent therapeutic efficacy in chronic CRSC. Carbonic anhydrase inhibitors may therefore represent one of the few remaining therapeutic options in such settings. However, larger prospective cohorts are needed to determine their efficacy and establish their potential role in the management of chronic CRSC. Although our study is limited to the observation of a single case, the favorable anatomical outcome observed is encouraging and may provide a basis for further investigation of this therapeutic approach. This case illustrates the importance of follow-up even when active leakage is absent due to the risk of progressive atrophic degeneration.

## References

[1] Nicholson B, Noble J, Forooghian F, Meyerle C (2013). Central serous chorioretinopathy: update on pathophysiology and treatment. Surv Ophthalmol.

[2] Daruich A, Matet A, Marchionno L, De Azevedo JD, Ambresin A, Mantel I, Behar-Cohen F (2017). Acute central serous chorioretinopathy: factors influencing episode duration. Retina.

[3] Daruich A, Matet A, Dirani A (2015). Central serous chorioretinopathy: recent findings and new physiopathology hypothesis. Prog Retin Eye Res.

[4] Fung AT, Yang Y, Kam AW (2023). Central serous chorioretinopathy: a review. Clin Exp Ophthalmol.

[5] Kaye R, Chandra S, Sheth J, Boon CJF, Sivaprasad S, Lotery A (2020). Central serous chorioretinopathy: an update on risk factors, pathophysiology and imaging modalities. Prog Retin Eye Res.

[6] Spaide RF, Gemmy Cheung CM, Matsumoto H (2022). Venous overload choroidopathy: a hypothetical framework for central serous chorioretinopathy and allied disorders. Prog Retin Eye Res.

[7] Warrow DJ, Hoang QV, Freund KB (2013). Pachychoroid pigment epitheliopathy. Retina.

[8] Pang CE, Freund KB (2015). Pachychoroid neovasculopathy. Retina.

[9] Phasukkijwatana N, Freund KB, Dolz-Marco R (2018). Peripapillary pachychoroid syndrome. Retina.

[10] Siedlecki J, Schworm B, Priglinger SG (2019). The pachychoroid disease spectrum-and the need for a uniform classification system. Ophthalmol Retina.

[11] Kitzmann AS, Pulido JS, Diehl NN, Hodge DO, Burke JP (2008). The incidence of central serous chorioretinopathy in Olmsted County, Minnesota, 1980-2002. Ophthalmology.

[12] Liew G, Quin G, Gillies M, Fraser-Bell S (2013). Central serous chorioretinopathy: a review of epidemiology and pathophysiology. Clin Exp Ophthalmol.

[13] Mohabati D, van Rijssen TJ, van Dijk EH (2018). Clinical characteristics and long-term visual outcome of severe phenotypes of chronic central serous chorioretinopathy. Clin Ophthalmol.

[14] Dansingani KK, Gal-Or O, Sadda SR, Yannuzzi LA, Freund KB (2018). Understanding aneurysmal type 1 neovascularization (polypoidal choroidal vasculopathy): a lesson in the taxonomy of 'expanded spectra' - a review. Clin Exp Ophthalmol.

[15] El Ameen A, Cohen SY, Semoun O (2015). Type 2 neovascularization secondary to age-related macular degeneration imaged by optical coherence tomography angiography. Retina.

[16] van Rijssen TJ, van Dijk EHC, Yzer S (2019). Central serous chorioretinopathy: towards an evidence-based treatment guideline. Prog Retin Eye Res.

[17] Moreno-Morillo FJ, Fernández-Vigo JI, Güemes-Villahoz N, Burgos-Blasco B, López-Guajardo L, Donate-López J (2021). Update on the management of chronic central serous chorioretinopathy. Arch Soc Esp Oftalmol (Engl Ed).

[18] van Rijssen TJ, van Dijk EH, Tsonaka R (2022). Half-dose photodynamic therapy versus eplerenone in chronic central serous chorioretinopathy (SPECTRA): a randomized controlled trial. Am J Ophthalmol.

[19] van Dijk EH, Fauser S, Breukink MB (2018). Half-dose photodynamic therapy versus high-density subthreshold micropulse laser treatment in patients with chronic central serous chorioretinopathy: the PLACE trial. Ophthalmology.

[20] Wuarin R, Kakkassery V, Consigli A (2019). Combined topical anti-inflammatory and oral acetazolamide in the treatment of central serous chorioretinopathy. Optom Vis Sci.

[21] Lotery A, Sivaprasad S, O'Connell A (2020). Eplerenone for chronic central serous chorioretinopathy in patients with active, previously untreated disease for more than 4 months (VICI): a randomised, double-blind, placebo-controlled trial. Lancet.

[22] Ji S, Wei Y, Chen J, Tang S (2017). Clinical efficacy of anti-VEGF medications for central serous chorioretinopathy: a meta-analysis. Int J Clin Pharm.

